# Candidates for chemosensory genes identified in the Chinese citrus fly, *Bactrocera minax,* through a transcriptomic analysis

**DOI:** 10.1186/s12864-019-6022-5

**Published:** 2019-08-14

**Authors:** Zhongzhen Wu, Cong Kang, Mengqiu Qu, Junlong Chen, Mingshun Chen, Shuying Bin, Jintian Lin

**Affiliations:** 1 Guang Zhou City Key Laboratory of Subtropical Fruit Tree Outbreak Control, Zhongkai University of Agriculture and Engineering, Guangzhou, 510225 People’s Republic of China; 2 Department of Entomology, Kansas State University, Manhattan, KS 66506 USA

**Keywords:** *Bactrocera minax*, Gene expression, Transcriptome, Chemosensory genes

## Abstract

**Background:**

The males of many *Bactrocera* species (Diptera: Tephritidae) respond strongly to plant-derived chemicals (male lures) and can be divided into cue lure/raspberry ketone (CL/RK) responders, methyl eugenol (ME) responders and non-responders. Representing a non-responders, *Bactrocera minax* display unique olfactory sensory characteristics compared with other *Bactrocera* species. The chemical senses of insects mediate behaviors that are associated with survival and reproduction. Here, we report the generation of transcriptomes from antennae and the rectal glands of both male and female adults of *B. minax* using Illumina sequencing technology, and annotated gene families potentially responsible for chemosensory.

**Results:**

We developed four transcriptomes from different tissues of *B. minax* and identified a set of candidate genes potentially responsible for chemosensory by analyzing the transcriptomic data. The candidates included 40 unigenes coding for odorant receptors (ORs), 30 for ionotropic receptors (IRs), 17 for gustatory receptors (GRs), three for sensory neuron membrane proteins (SNMPs), 33 for odorant-binding proteins (OBPs), four for chemosensory proteins (CSPs). Sex- and tissue-specific expression profiles for candidate chemosensory genes were analyzed via transcriptomic data analyses, and expression profiles of all ORs and antennal IRs were investigated by real-time quantitative PCR (RT-qPCR). Phylogenetic analyses were also conducted on gene families and paralogs from other insect species together.

**Conclusions:**

A large number of chemosensory genes were identified from transcriptomic data. Identification of these candidate genes and their expression profiles in various tissues provide useful information for future studies towards revealing their function in *B. minax.*

**Electronic supplementary material:**

The online version of this article (10.1186/s12864-019-6022-5) contains supplementary material, which is available to authorized users.

## Background

Olfaction is of vital significance for survival and reproduction of insects, and associated with mediating insect behaviors, such as host-identification, predator-avoidance, mating, and oviposition [1]. Environmental chemicals elicit physiological and behavioural responses by means of olfactory signal transduction, in which chemical signals are converted to electrical signals that can be interpreted by the olfactory nervous system [2, 3]. The initial step in odor detection starts with the binding of odour molecules to the odorant receptors that are bound to dendrites of olfactory receptor neurons (ORNs) in antennae [4, 5]. The whole process requires several families of chemosensory genes, including genes coding for odorant receptors (ORs), ionotropic receptors (IRs), gustatory receptor (GRs), sensory neuron membrane proteins (SNMPs), odorant-binding proteins (OBPs) and chemosensory proteins (CSPs) [6, 7]. Insect OBPs bind and transport odorant molecules across the aqueous lymph, then release the odorants and activate ORs in the dendrites of ORNs [1]. CSPs are homologous to OBPs [8] and are believed to have functions similar to that of OBPs [9, 10]. Insect ORs are heterodimers composed of at least two proteins, a highly conserved OR co-receptor (ORco) acting as an ion channel, and a specific OR subunits (ORx) that determines ligand specificity [11, 12]. An OR/ORco complex functions as a ligand-gated ion channel and is activated by odorant molecule. A chemical signal is then transformed into an electric signal that is transmitted to a higher-ordered neural center [2, 3]. IRs are a family of various ionotropic glutamate receptors. IRs exist as heteromeric complexes with one co-receptor IR (such as IR25a, IR8a or IR76b) in association with one or more ligand-specific IRs within a single ORN [13, 14]. The “antennal IRs” sub-family that is predominately or specifically expressed in antennae, was initially defined as another olfactory receptor [15]. However, recent functional studies indicate that antennal IRs have diverse functions (beyond chemosensation functions), including odour detection such as ammonia and amines [16], polyamines [17], acids [14, 18], sex pheromones [19], as well as gustation [20–23], thermosensation [24, 25] and hygrosensation [26]. GRs are another G-protein coupled receptor family that are distantly related to ORs, and are broadly expressed in the antennae, mouthparts, wings and ovipositor of the insects, which are generally tuned for tasting (bitter and sweet) [27–32] and carbon dioxide detection [33, 34]. SNMPs are transmembrane proteins and belong to the CD36 receptor family [35, 36]. The *Drosophila melanogaster* SNMP subtype SNMP1 is involved in pheromone reception, and is located in the dendritic membrane of pheromone-sensitive neurons, and triggers ligand delivery to a pheromone receptor [37–39].

The Chinese citrus fly, *Bactrocera minax* (Enderlein) (Diptera: Tephritidae), is one of the most devastating pests of citrus, and is distributed in the temperate areas of Asia including Nepal, India, Bhutan, and China [40, 41]. The males of many *Bactrocera* species (Diptera: Tephritidae) respond strongly to plant-derived chemicals (male lures) and broadly categorized into three groups of species based on the characteristics of their chemosensory: cue lure/raspberry ketone responders (CL/RK), methyl eugenol (ME) responders and non-responders [42, 43]. *B. minax* belongs to the last category, a non-responder. In terms of host range, *B. minax* is oligophagous, feeding on cultivated and wild species of citrus. During the long course of coevolution with its host plants, the olfaction system of *B. minax* is likely different from the CL/RK and ME responders, or polyphagous counterparts such as *B. dorsalis.* At the peripheral olfactory signaling, diversifying chemoreceptor gene families may allow the detection and differentiation of a wide array of host volatiles, therefore polyphagous insects could possess a diverse set of chemosensory receptors relative oligophagous [5, 44–46]. At present, little is known about the genes and molecular events involved in chemosensory in this representative *Bactrocera* species.

The objective of this study is to identify genes potentially involved in chemosensory following a transcriptomic approach. We generated transcriptomes from dissected antennae and rectal glands from both male and female adults. Rectal glands are involved in potential sex pheromone production in *Bactrocera* species [47, 48]. Moreover, members of the chemosensory multigene families are expressed in pheromone glands in Lepidoptera where they are involved in pheromone product process [49–53]. We used this approach to identify a set of candidate chemosensory genes comprising ORs, IRs, GRs, SNMPs, OBPs and CSPs. We constructed a comprehensive and comparative phylogenetic trees to examine the characteristics of *B. minax* chemosensory genes and their relationship to that of other insects. In addition, the sex- and tissue-specific expression profiles of chemosensory genes were determined via fragments per kilobase per million reads (FPKM) and real-time quantitative PCR (RT-qPCR). Our results should provide a basis for future studies to reveal olfactory receptive mechanisms for the olfactory system of *B. minax.*

## Results

### Transcriptome assembly

A total of 53.4, 53.4, 52.8 and 51.1 million raw reads were obtained by sequencing the libraries derived from dissected female antennae, male antennae, female rectal glands and male rectal glands, respectively. After trimming adaptor sequences, eliminating low quality reads, and removing contaminant sequences, 51.8, 51.8, 51.2 and 49.6 millions of clean reads were retained from these four transcriptomes, respectively. Combined trinity assembly of the clean reads generated 120,803 unigenes with a mean length of 717 bp, an N50 of 1306 bp, and an N90 of 267 bp. The number of unigenes longer than 1 Kb was 34,832, which was listed at Additional file 1: Table S1.

### Functional annotation of assembled unigenes

Annotation was conducted by BLASTx and BLASTn programs with e-value cut-off 10^− 5^. A total of 36,287 (30.03%) unigenes were annotated by searching against at least one of the databases. Specifically, 26,043 (21.55%) unigenes were annotated by blasting against the NCBI-non-redundant protein sequence (Nr) database, 18,005 (14.90%) unigenes against the NCBI-non-redundant nucleotide (Nt) database, 22,269 (18.43%) based on PFAM, 11,209 (9.27%) based on the Clusters of Orthologous Groups (KOG/COG) database, 16,147 (13.36%) by searching against the SwissPort database, 22,505 (18.62%) based on Gene Ontology (GO), and 9942 (8.22%) based on Kyoto Encyclopedia of Genes and Genomes (KEGG) (Additional file 2: Table S2).

Species with the highest proportion of similar genes were *B. dorsalis* (26.1%) followed by *B. cucurbitae* (21.9%), *B. oleae* (12.1%), *Ceratitis capitata* (4.3%) and *Rhagoletis zephyria* (3.4%) (Additional file 3: Figure S1A). GO analysis was used to categorize annotated genes into three functional categories: ‘biological process’, ‘cellular component’, and ‘molecular function’. In ‘biological process’, subcategories ‘cellular’, ‘single-organism’ and ‘metabolic’ contained the majority of the unigenes. In ‘cellular component’, the subcategories ‘cell’ and ‘cell part organelle’ contained the majority unigenes. In ‘molecular function’, the subcategories ‘binding’ and ‘catalytic activity’ were with the largest numbers of unigenes (Additional file 3: Figure S1B). Functional categories and pathways based on a KEGG analysis are given in Additional file 3: Figure S1C. The categories ‘signal transduction’, ‘translation’, ‘transport’ and ‘catabolism’ were on the top among the 32 categories in terms of the number of unigenes.

### Candidate odorant receptors

In this study, 40 putative OR unigenes were identified from sequencing the *B. minax* tissue-specific libraries. The proteins encoded by these OR unigenes belong to the receptor superfamily with 7 transmembrane domain receptors (the 7-transmembrane receptors superfamily). Among the transcripts corresponding to these OR unigenes, 37 encode full-length proteins with 306 to 417 amino acid residues with 4–8 transmembrane domains (TMDs). Other partial unigenes encoded proteins exhibiting overlapping regions with low sequence identity (Additional file 4: Table S3). One of the OR putative protein shares 99% identity to a co-receptor from *B. cucurbitae* (XP_011183998.1) and was named as BminORco. Other identified ORs from *B. minax* were also similar to reported ORs from *Bactrocera* species, with at least 60% amino acid sequence identity.

**Fig. 1:**
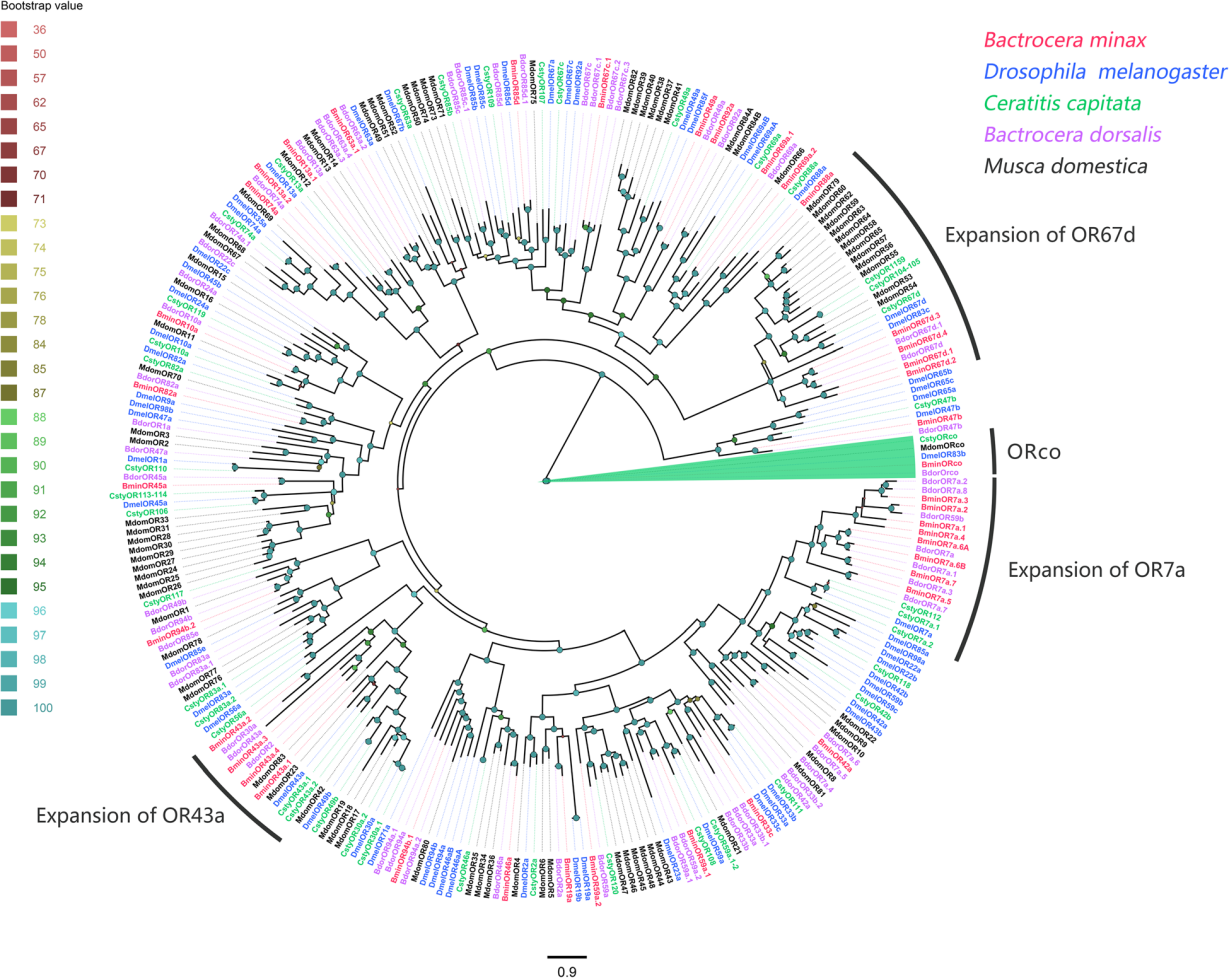
Phylogenetic tree of candidate *Bactrocera minax* ORs and homologs from other Dipterans. The distance tree was rooted by the conservative ORco gene orthologues. Bootstrap values are shown. The ORco clade, OR67d clade, OR43a clade and OR7a clade are shown. Sequences from species in this phylogeny include *Bactrocera minax* (Bmin, hot pink), *Drosophila melanogaster* (Dmel, bule), *Ceratitis capitate* (Ccap, spring green), *Bactrocera dorsalis* (Bdor, purple), and *Musca domestica* (Mdom, black)

A maximum likelihood tree was created using IQ-TREE (version 2.1.7) with best-fitting substitution-model. The phylogenetic tree was generated using our identified putative OR proteins along with a data set containing representative ORs from four other Dipterans; *D. melanogaster*, *C. capitate*, *B. dorsalis* and *Musca domestica* (Fig. 1). The vast majority of BminORs were clustered with orthologues from other species. A clade containing OR7a homologs and DmelOR7a, was greatly expanded in *B. minax* as well as in *B. dorsalis*. Eight BminORs (BminOR7a.1, OR7a.2, OR7a.3, OR7a.4, OR7a.5, OR7a.6A, OR7a.6B and OR7a.7) were clustered with DmelOR7a. Moderate expansion of clades containing OR43a and OR67d was also observed in *B. minax*. Four BminORs (BminOR43a.1, OR43a.2, OR43a.3 and OR43a.4) were clustered with DmelOR43a from *Drosophila*, and four BminORs (BminOR67d.1, OR67d.2, OR67d.3 and OR67d.4) were clustered with DmelOR67d.

### Candidate ionotropic receptors

Thirty putative iGluR/IR unigenes were identified from the *B. minax* samples. Of the iGluR/IR transcripts corresponding to these unigenes, 19 encoded full-length proteins with at least 503 amino acid residues. Amino acid sequences encoded by these transcripts share high sequence similarity to ligand-gated cation channels with three full or partial TMDs (M1, M2 and M3) and a ligand-binding domain (S1 and S2) (Additional file 4: Table S3), which was characteristic of insect iGluRs/IRs [15].

**Fig. 2:**
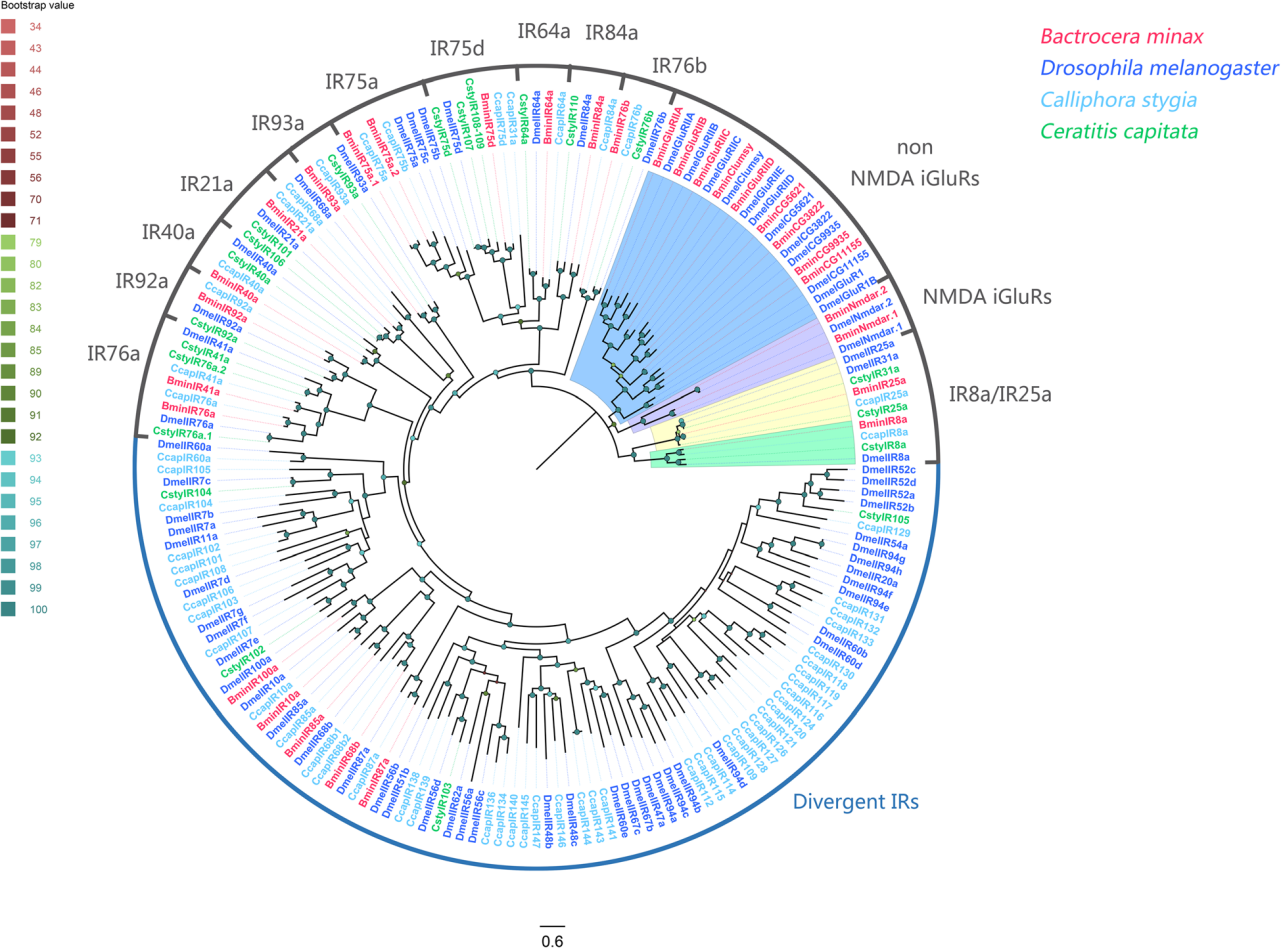
Phylogenetic tree of candidate *Bactrocera minax* IRs and other Dipteran IRs. The distance tree was rooted by the conservative IR25a/IR8a gene orthologues. Bootstrap values are shown. The IR25a/IR8a clade, iGluRs clade and some antennal-associated orthologue clade are shown. This tree was constructed using the following species: *Bactrocera minax* (Bmin, hot pink), *Drosophila melanogaster* (Dmel, bule), *Calliphora stygia* (Csty, light blue), *Ceratitis capitate* (Ccap, spring green)

Distinct clades were observed in a phylogenetic tree generated with our identified sequences and paralogs from other species including *D. melanogaster*, *C. stygia* and *C. capitate* IRs (Fig. 2). Identified candidate antennal IRs (14) were clustered with previously reported “antennal” orthologues BminIR8a, IR25a, IR21a, IR40a, IR41a, IR64a, IR75a.1, IR75a.2, IR75d, IR76a, IR76b, IR84a, IR92a and IR93a; and were well separated from those non-NMDA iGluRs, NMDA iGluRs and divergent IRs clades. Interestingly, a usually conserved “antennal” orthologue, IR68a, was absent from *B. minax*. Instead, two IR75a orthologues (BminIR75a.1 and IR75a.2) was found from *B. minax*.

### Candidate gustatory receptors

**Fig. 3:**
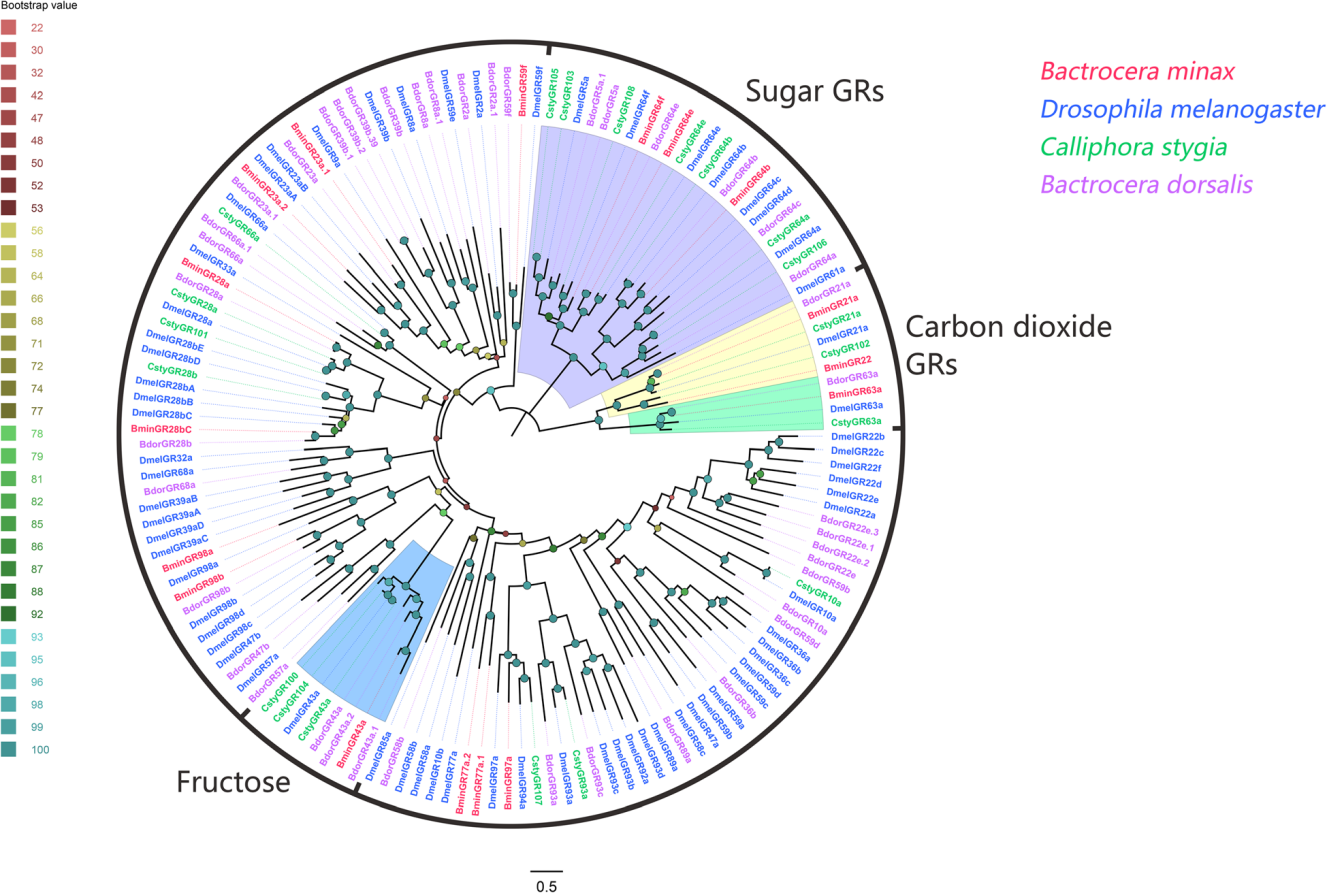
Phylogenetic tree of candidate *Bactrocera minax* GRs and other Dipteran GRs. The distance tree was rooted by the conservative carbon dioxide GRs gene orthologous. Bootstrap values are shown. The carbon dioxide GRs clade and sugar GRs clade are shown. This tree was constructed using the species *Bactrocera minax* (Bmin, hot pink), *Drosophila melanogaster* (Dmel, bule), *Calliphora stygia* (Csty, spring green), *Bactrocera dorsalis* (Bdor, purple)

Seventeen GR candidates were identified from *B. minax*, and all of them encode full-length proteins with 4–8 TMDs (Additional file 4: Table S3). Functions of GRs identified from *B. minax* could be inferred from their phylogenetic relationship with GRs previously well characterized from other dipteran species (Fig. 3). BminGR21a, GR22 and GR63a were clustered with carbon dioxide GRs (DmelGR21a and DmelGR63a) [33, 34]. BminGR43a was clustered with the *Drosophila* fructose receptor DmelGR43a [54]. Three other GRs (BminGR64b, GR61e and GR64f) were clustered with *Drosophila* sugar receptors (DmelGR64b, GR61e and GR64f), respectively [29–31, 55, 56].

### Candidate sensory neuron membrane proteins

**Fig. 4:**
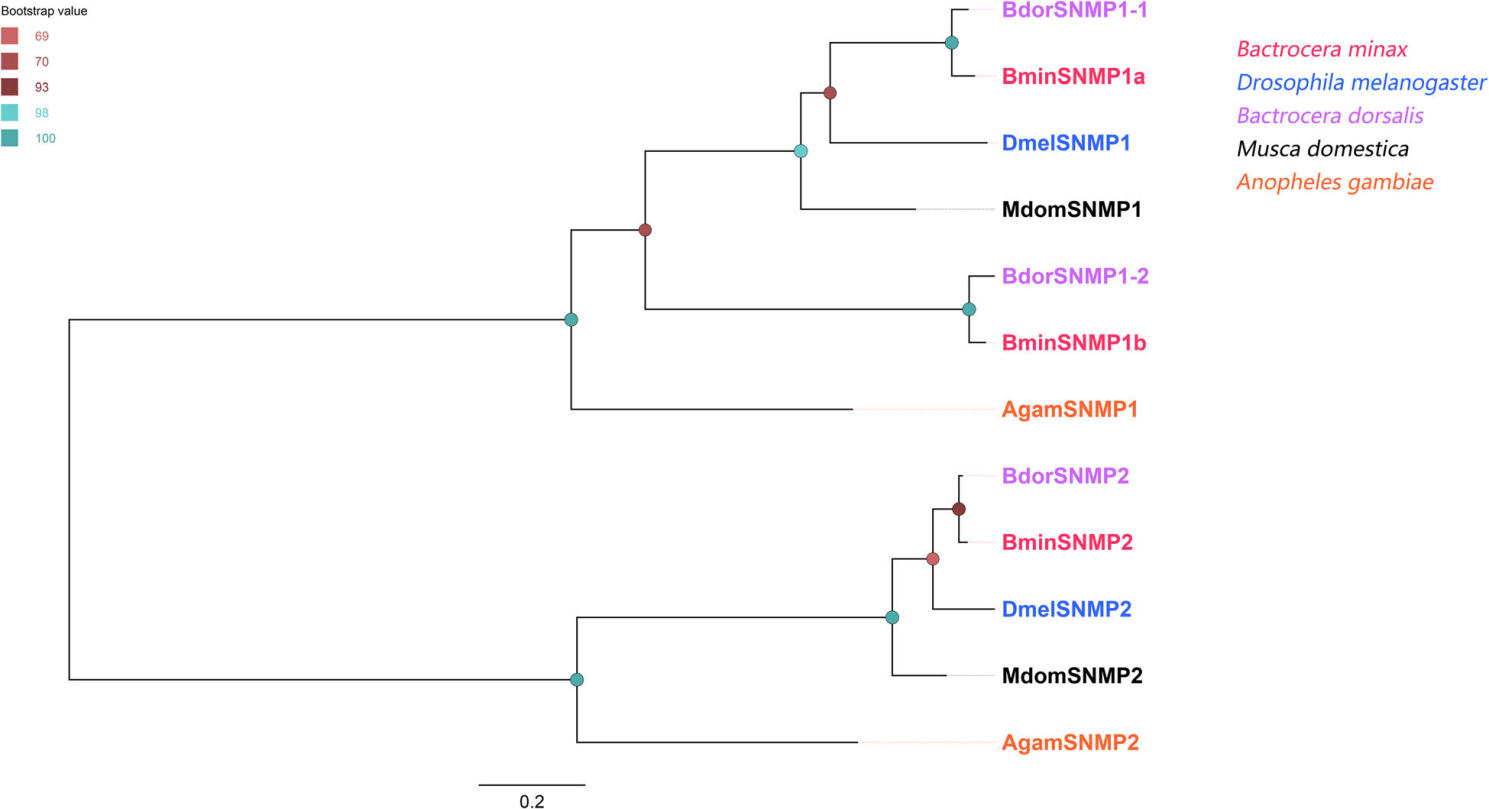
Phylogenetic tree of candidate *Bactrocera minax* SNMPs and other Dipteran SNMPs. Bootstrap values are shown. The species used to construct tree including *Bactrocera minax* (Bmin, hot pink), *Drosophila melanogaster* (Dmel, bule), *Bactrocera dorsalis* (Bdor, purple), *Musca domestica* (Mdom, black) and *Anopheles gambiae* (Agam, orange)

Three unigenes were found to encode full-lengthSNMPs with two TMDs were identified named BminSNMP1a, BminSNMP1b and BminSNMP2 (Additional file 4: Table S3). BminSNMP1a and BminSNMP1b were clustered with the *Drosophila* SNMP1, a protein required for correct pheromone detection [37, 38, 57, 58], while BmelSNMP2 clustered with other insect SNMP2 orthologues (Fig. 4).

### Candidate odorant-binding proteins

**Fig. 5:**
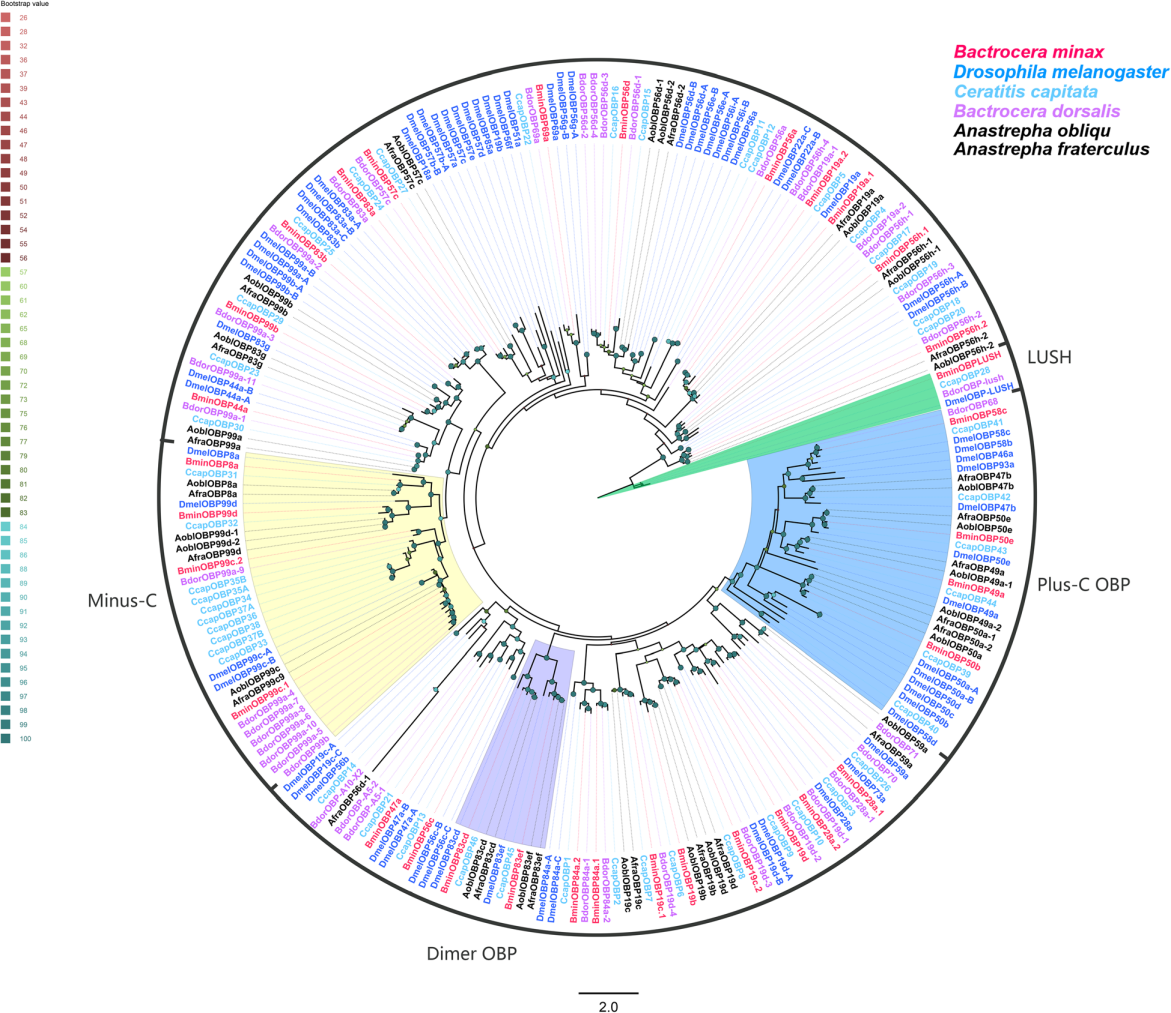
Phylogenetic tree of candidate *Bactrocera minax* OBPs and other Dipteran OBPs. The distance tree was rooted by lush gene orthologous. Bootstrap values are shown. The Classic OBPs clade, Plus-C OBPs clade, Minus-C OBPs and Dimer OBPs clade are shown. The species used to construct tree include *Bactrocera minax* (Bmin, hot pink), *Drosophila melanogaster* (Dmel, bule), *Calliphora stygia* (Csty, light blue), *Ceratitis capitate* (Ccap, spring green), *Bactrocera dorsalis* (Bdor, purple), *Episyrphus balteatus* (Ebal, black) and *Eupeodes corollae* (Eup, black)

A total of 33 OBP unigene were identified from the *B. minax* transcriptomes and all the identified unigenes encode full-length proteins. Except two (BminOBP50e and BminOBP57c), all predicted proteins have a signal peptide sequence (Additional file 4: Table S3). Among them, 23 Classic OBPs have six conserved cysteine residues, four Minus-C OBPs (BminOBP8a, OBP99c.1, OBP99c.2 and OBP 99d) have only four cysteine residues (C2 and C5), whereas four Plus-C OBPs (BminOBP49, OBP50b, OBP50e and OBP58c) have 4–6 more cysteine residues in addition to the six conserved cystteines. BminOBP83cd and OBP83ef were predicted to be Dimer OBPs with two six-cysteine signatures (Additional file 5: Figure S2). Phylogenetic tree of the identified OBPs with orthologs from other dipterans assigned *B. minax* OBPs to Plus-C, Minus-C and Dimer groups, and the remaining were assigned to the Classic groups (Fig. 5).

### Candidate chemosensory proteins

**Fig. 6:**
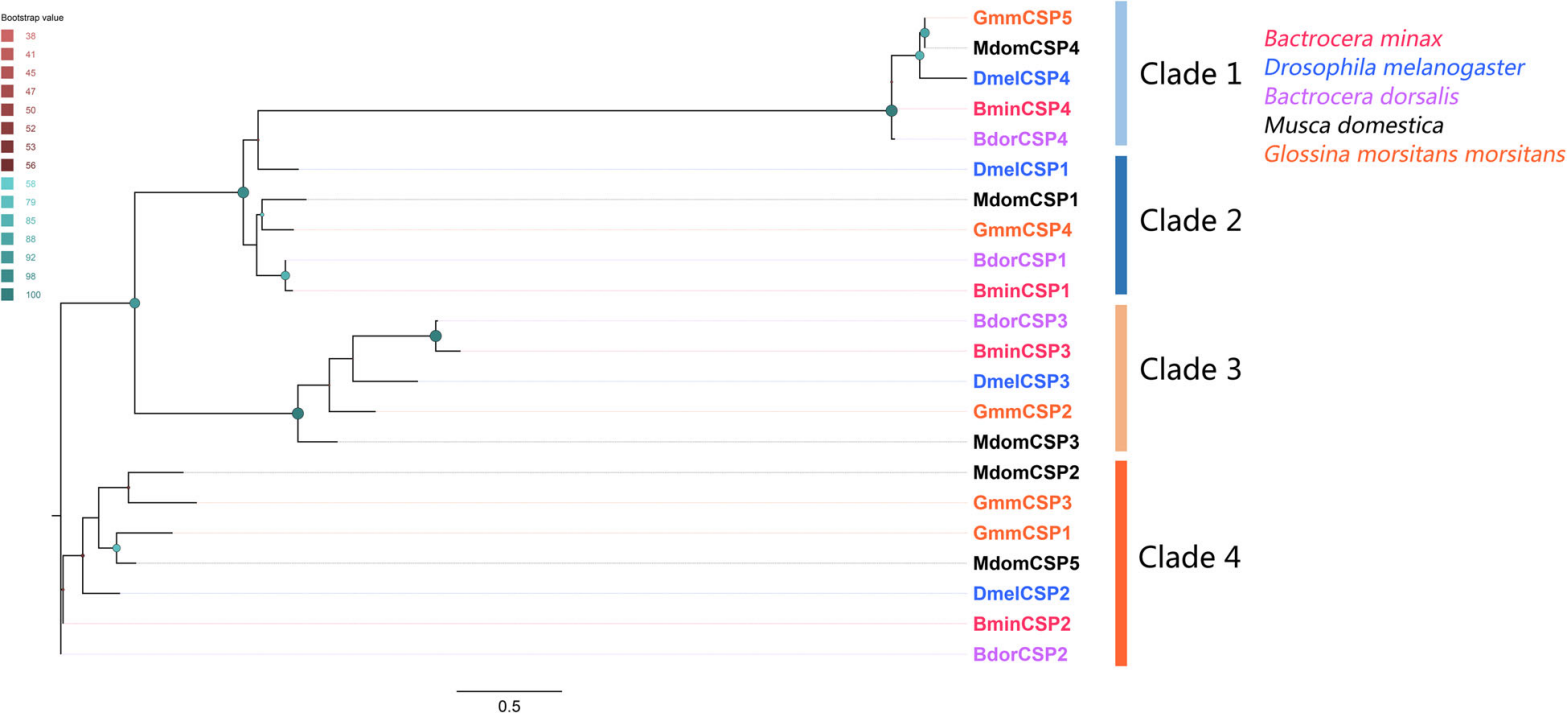
Phylogenetic tree of candidate *Bactrocera minax* CSPs and other Dipteran CSPs. Bootstrap values are shown. The four sub-clades (1–4) are shown. The species used to construct tree include *Bactrocera minax* (Bmin, hot pink), *Drosophila melanogaster* (Dmel, bule), *Bactrocera dorsalis* (Bdor, purple), *Musca domestica* (Mdom, black) and *Glossina morsitans morsitans* (Gmm, orange)

Four unigenes encoding CSPs were identified from the *B. minax* transcriptomes and all of them encode full-length proteins (Additional file 4: Table S3). Predicted proteins contain four highly conserved cysteine residues and a signal peptide (Additional file 6: Figure S3). A phylogenetic analysis assigned each of the identified CSPs into four distinct clades together with homologs from other dipterans (Fig. 6).

### FPKM and differentially expressed genes

Transcript abundance of the identified ORs, IRs, GRs, SNMPs, OBPs and CSPs was initially estimated based on their FRKM values (Additional file 7: Table S4). Transcript abundance of ORs, IRs and GRs was relatively low in antennae compared with that of SNMPs, OBPs and CSPs. Among putative BminORs, the co-receptor (ORco) exhibited the highest level of transcript abundance (female: 380.43 and male: 390), compared with other ORs, which ranged from 0.39 to 106. The RPKM values for putative BminIRs ranged from 5.36 to 133. BminIR93a exhibited the highest transcript abundance, followed by BminIR25a, BminIR8a and BminIR76b. The overall expression levels of putative GRs were relatively low. Among them, BminGR21a had significantly higher transcript abundance than that of other BminGRs. Among the identified BminOBPs, BminOBP28a.2 showed the highest transcript abundance, followed by BminOBP83b and BminOBP83a. For the identified SNMPs and CSPs, BminSNMP1a and BminCSP2 exhibited the highest transcript abundance.

**Fig. 7:**
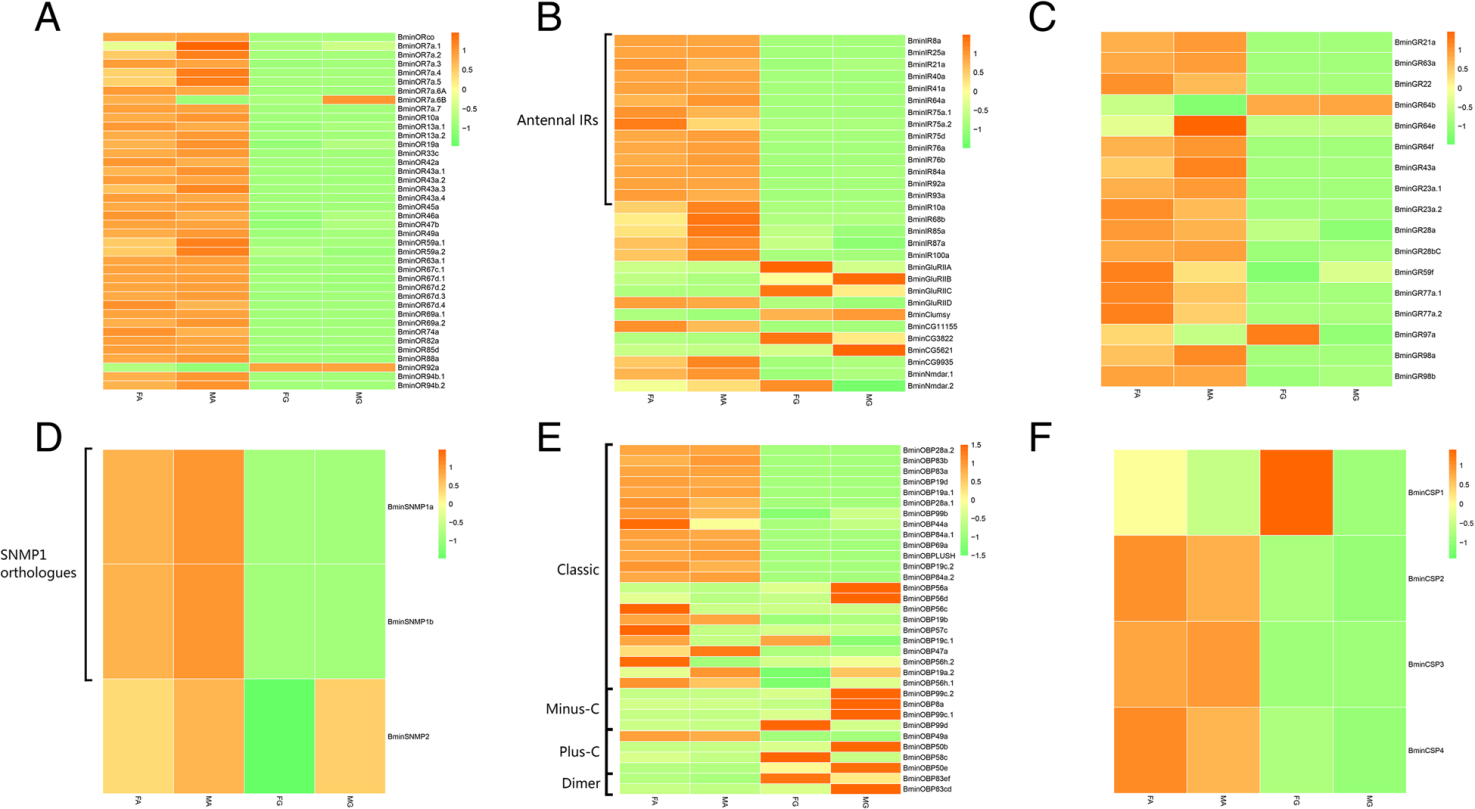
Tissue- and sex-specific expression profiles of chemosensory genes in antennae and rectal glands. Expression levels of the chemosensory genes in the four transcriptomes represented as heat plots based on log-transformed FPKM values. **a** ORs; **b** IRs; **c** GRs; **d** SNMPs; **e** OBPs and **f** CSPs. Abbreviations: FA, female antennae; MA, male antennae; FG, female rectal glands; MG; male rectal glands

Figure 7 provides more details on gene expression of all the identified genes among different tissues and sexes using a heat plot. Of the 40 ORs, 38 exhibited high transcript abundance in antennae from both sexes. The remaining two ORs (BminOR7a.6B and OR92a) showed higher transcript abundance in rectal glands than in antennae in both males and females. For IRs, all antennal IRs were specifically expressed in antennae. For GRs, BminGR64b and GR97a exhibited higher transcript abundance in rectal glands, whereas others showed higher abundance in antennae. For SNMPs, BminSNMP1a and SNMP1b exhibited higher abundance in antennae. For OBPs, 19 Classic OBPs and one Plus-C (BminOBP49a) exhibited higher abundance in antennae than that in rectal glands. For CSPs, BminCSP2, CSP3 and CSP4 showed higher abundance in antennae. In terms of sexes, none of the GRs, SNMPs, OBPs and CSPs showed a drastic difference in transcript abundance between females and males.

### Real-time quantitative PCR analysis

**Fig. 8:**
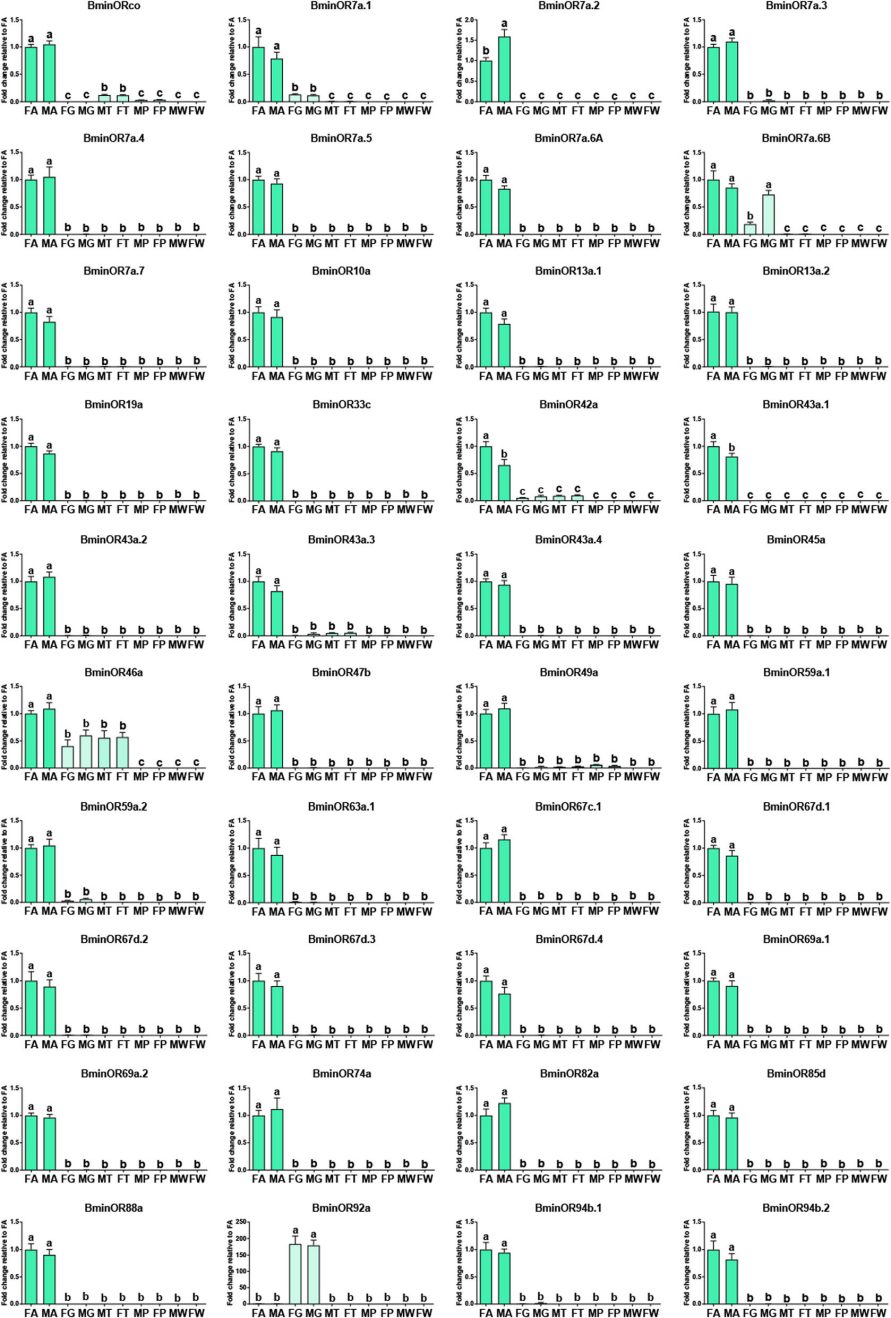
Relative transcript levels of all putative ORs in the different tissues, using RT-qPCR. Abbreviations: FA, female antennae; MA, male antennae; FG, female rectal glands; MG; male rectal glands; MT, male foreleg tarsi; FT, female foreleg tarsi; MP, male proboscises; FP, female proboscises; MW, male wings; FW, female wings. The relative expression level is indicated as mean ± SE (*n* = 3). Standard error is represented by the error bar, and different letters indicate significant differences between tissues (*p* < 0.05, ANOVA, HSD)

**Fig. 9:**
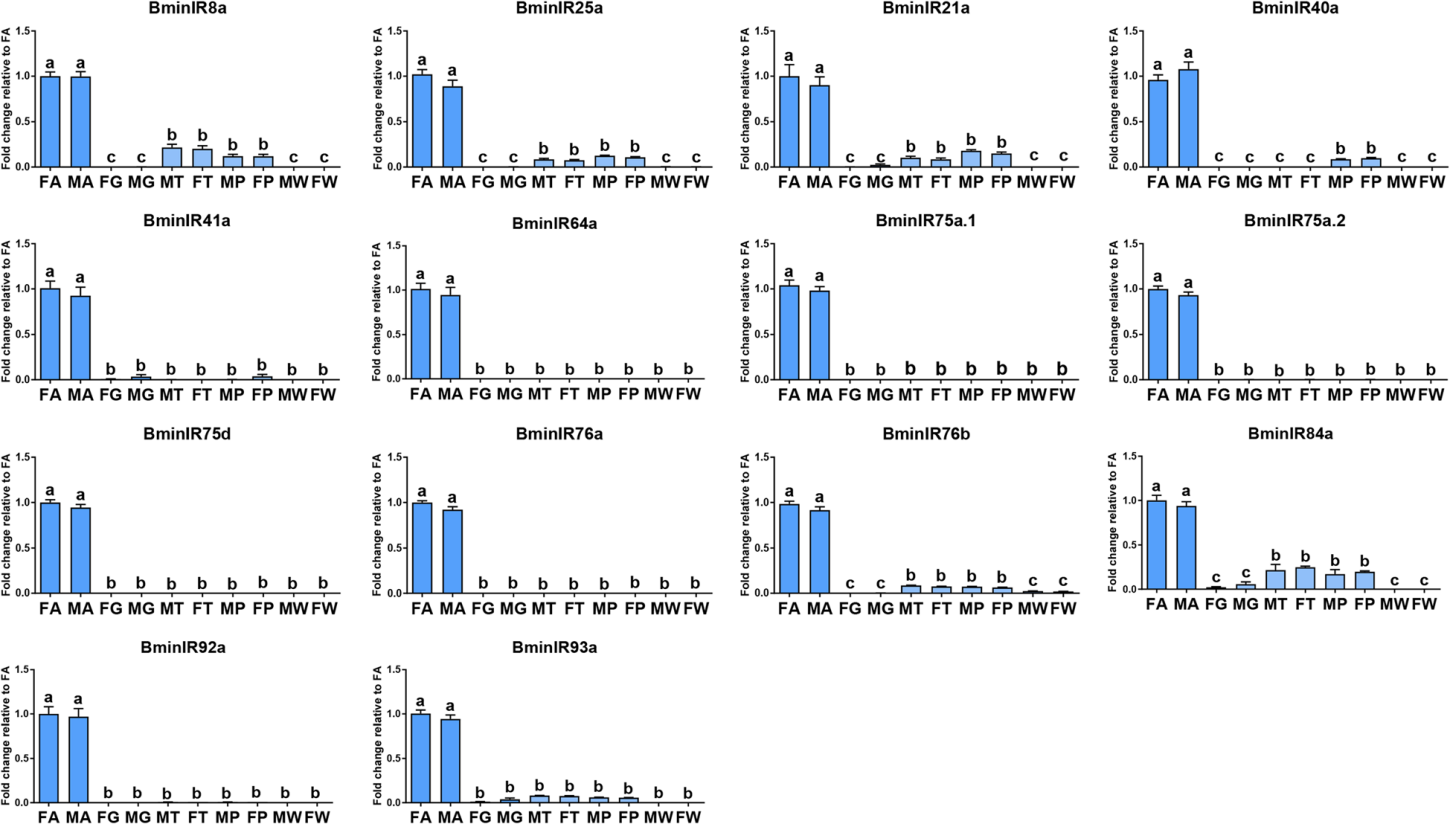
Relative transcript levels of all putative antennal IRs in the different tissues, using RT-qPCR. Abbreviations: FA, female antennae; MA, male antennae; FG, female rectal glands; MG; male rectal glands; MT, male foreleg tarsi; FT, female foreleg tarsi; MP, male proboscises; FP, female proboscises; MW, male wings; FW, female wings. The relative expression level is indicated as mean ± SE (*n* = 3). Standard error is represented by the error bar, and different letters indicate significant differences between tissues (*p* < 0.05, ANOVA, HSD)

Selected genes were further analyzed via RT-qPCR in different tissues. Transcript levels of all 40 ORs and 14 antennal IRs were successfully detected through RT-qPCR (Figs. 8 and 9). RT-qPCR revealed that a large number of ORs were antenna-predominant except for BminOR7a.6B, and OR92a, which exhibited higher transcript abundance in rectal glands. Among antenna-predominant ORs, all ORs but three (BminOR7a.2, OR42a and OR43a.1) were equally expressed in both males and females, and BminOR7a.2 was more abundant in males, while BminOR42a and OR43a.1 was more abundant in females. For the antennal IRs, all were specifically expressed in antennae, and no significant differences in transcript abundances were observed between males and females. Overall the RT-qPCR data mirror a similar trend with the corresponding FPKM values (Additional file 7: Table S4).

## Discussion

Based on olfactory responses to plant attractants, *Bactrocera* species can be categorized into CL/RK-, ME - and non-responders. *B. minax* is a non-responder based on previous reports. Biologically, *B. minax* is an oligophagous insect that oviposits only into the fruit of Citrus species. This is different from most of *Bactrocera* species such as *B. dorsalis*. The difference in oviposition behavior may reflect difference in olfactory sensation specificity with *B. minax*. Prior to this study, the chemosensory gene families had been identified from other *Bactrocera* species that are highly polyphagous [59–61]. Here, we present the results of genetic and phylogenetic analyses of putative chemosensory genes in an oligophagous *Bactrocera* species to examine the similarities and differences of molecular components in chemosensory pathways. We further analyzed the expression profiles of identified chemosensory genes in an olfactory (antennae) and a non-olfactory tissue (rectal glands) to identify olfaction-specific genes for future functional studies.

The numbers of putative OR transcripts identified in *B. minax* (40 in the antennae) were close to the number (43) identified in *B. dorsalis* antennae [60]*.* This suggests that *Bactrocera* ORs shows conservation in gene numbers. Even the overall numbers of genes are comparable, there are specific differences in gene compositions among *Bactrocera* species. Compared to homologous ORs previously reported for *B. dorsalis*, there were lower number in *B. minax*, i.e., OR67c, OR85d, OR63a, OR59a (Fig. 1). This suggests a possible link between olfactory perception and host adaptation, *B. minax* have relatively narrow host ranges, which limited to several Citrus species. Notably, there is a great expansion of ORs with similarity to the aggregation/egg-laying decisions-linked receptor (OR7a) [62], which is putatively responsible to the pheromone benzaldehyde (OR43a) [63], and the pheromone cis-vaccenyl acetate receptor (OR67d) [64]. This may imply that the gene expansion is likely to enhance their food and pheromone-odor perception. Alternatively, it may require a set of homologous ORs to detect specific odorants or a combination of similar odorants. More members of the OR67d family have been observed in *B. minax*, which may suggest the importance of enhancing their pheromone perception for mating. BminOR42a and OR43a.1 were expressed predominantly in females, suggesting that these two ORs may be involved in recognition of plant volatiles for oviposition. On the other hand, BminOR7a.2 was predominantly expressed in the antennae of males, and may be involved in female pheromone perception. Sex-biased expression of these ORS appears specific to *B. minax* since no sex-biased expression of counterparts was observed in *B. dorsalis* ORs [65]. Additionally, our results found that BminOR92 have up-regulated in expression in the rectal glands, which differ from most of the ORs up-regulated in the antennae of insect, and may have different physiological functions, such as sex pheromone production.

Members in the IR family identified here are relatively conserved, especially with respect to those antennal IR receptors. The number of antennal IR genes expressed in *B. minax* antennae (14) is similar to that in *D. melanogaster* (14) and other Dipterans. Our phylogenetic analysis indicated that 14 antennal IRs in *B. minax* have orthologs from other Dipterans. According to functional studies of antennal IRs in *D. melanogaster*, IR92a has a narrow tuning function for sensitivity to ammonia and amines [16]. A combination of IR76b/IR41a is for polyamine sensing [17], IR75a/IR8a for acetic acid sensing [66], DmelIR84a/8a for promoting male courtship via phenylacetaldehyde and phenylacetic acid [19], IR64a/IR8a for acids sensing [14, 18], IR21a/IR25a for cool temperatures sensing [25], a complex of IR93a with IR25a and IR40a/IR68a for moisture detection [24, 26]. The IRs orthologs in *B. minax* might play the same role in sensory functions. In addition to these IRs similar to known *Drosophila* antennal IRs, we also identified IR75d in *B. minax* antennae, its orthologs in other species have not been functionally verified.

Although transcript abundance of BminGRs is low in analyzed tissues, the identified unigenes in *B. minax* all encode full-length proteins, indicating our transcriptomes were in high-quality. One *B. minax* GR, BminGR22, a homolog of GR21a that mediates CO_2_ recognition, was also highly expressed in antennae. It is not clear if BminGR22 may also play a role in recognizing some fruit cues even though its high expression in antennae suggests biological significance in antennal sensing. Further functional analyses are required to identify its physiological roles. In addition, four *B. minax* GRs, BminGR43a, GR64b, GR64e and GR64f, were separately clustered with a fructose-detecting GR and several other sugar-detecting GRs from *Drosophila*, indicating that they may perform similar functions.

In *D. melanogaster*, SNMP1 subfamily is antenna-specific and associated with pheromone-sensitive ORNs, and is essential for the perception of the pheromone cis-vaccenyl acetate. In contrast, the general mechanism for SNMP2 functions remain unclear. In the present study, three BminSNMPs were identified in *B. minax*. Among these, two SNMP1 homologs (BminSNMP1a and 1b) exhibited a clear antenna-predominant expression, suggesting that BminSNMP1a and BminSNMP1b may be associated with pheromone reception.

Two antenna-specific OBPs (BminOBP83a and OBP83b) were clustered with the OBP83a orthologs form *D. melanogaster*, *C. capitate*, and *B. dorsalis*, which were exclusively expressed in antennae, and were reported to play crucial roles in olfactory perception, such as pheromone components perception in *C. capitate* [67], and attractant detection in *B. dorsalis* [68]*.* Furthermore, BminOBP83a and OBP83b exhibited the second- and third-highest transcript abundance in antennae among the OBP family members, suggesting that it may be associated with odorant perception.

In the antennae of *B. minax*, we identified 4 CSPs with similarity to homologues from *B. dorsalis* and *D. melanogaster*. BminCSP2 was antenna-specific, suggesting that it may play a role in chemoreception associated with antifeedants [69]. Further investigations are needed to reveal the specific functions of BminCSP2.

## Conclusions

In conclusion, we identified an extensive set of candidate genes that may be related to odorant perception in *B. minax* by analyzing transcriptomic sequence data. As the first step towards understanding gene functions, we conducted a comprehensive and comparative phylogenetic analysis and examined OR and antennal IR gene transcription patterns. Further analysis is needed to explore the function of these genes using integrated functional studies.

## Methods

### Ethics statement

The Chinese citrus fly, *B. minax* larvae collections were made with the direct permission of the owners of the orchards [Yichang district (30.6943° N, 111.2807° E) of Hubei province] and *B. minax* culture was maintained in our laboratory as mentioned below. We reaffirm that none of the *B. minax* collections were from National Parks or protected wilderness areas. Besides, *B. minax* are definitely not an endangered species.

### Insect rearing and tissue collection

Fallen oranges infested with *B. minax* larvae were collected from citrus orchards in Yichang district, Hubei province, China, in late October 2016. In laboratory, hundreds of larvae were allowed to pupate into sand with subsequent adult emergence into big mesh cages supplied with 5% sugar water and brewer’s yeast. The rearing conditions were as follows: temperature 25 ± 1°С, relative humidity 70 ± 10%, and photoperiod 14 h light: 10 h dark. The antennae (300 pairs of each sex) and rectal glands (50 of each sex) were separately excised from 5-day-old adults, and immediately frozen and stored in liquid nitrogen until use.

### cDNA library construction and transcriptome analysis

Total RNA of female antennae above was separately extracted using TRIzol reagent (Invitrogen, Carlsbad, CA, USA) following the manufacturer’s instructions. RNA integrity was determined with an Agilent Bioanalyzer 2100 system (Agilent Technologies Inc., CA, USA). RNA concentration and purity was measured on a Nanodrop ND-2000 spectrophotometer (NanoDrop Technologies Inc., Wilmington, DE). Three micrograms of total RNA per sample was used for cDNA library construction. cDNA library was prepared using Illumina’s sample preparation instructions (Illumina, San Diego, CA). The library was then sequenced on the Illumina HiSeq2500 platform (Illumina, San Diego, CA, United States) to obtain paired-end reads (150 bp).

Raw reads were processed to remove unknown (poly-N) or low-quality and adaptor sequences using Trimmomatic to obtain the clean data [70]. Trinity de novo program (Version: r20140413p1) with default parameters was used to assemble the clean reads. Redundant sequences were removed to obtain unigenes by means of selecting longest transcript contigs.

### Functional annotation

The assembled unigenes were annotated by BLAST-searching databases with (e-value cut-off <1e− 5). Databases used for annotation include the non-redundant protein sequence (Nr), non-redundant nucleotide (Nt), Pfam, Clusters of Orthologous Groups (KOG/COG), Swiss-Prot, Gene Ontology (GO) and Kyoto Encyclopedia of Genes and Genomes (KEGG) databases.

### Identification of chemosensory genes

To identify candidate chemosensory genes (ORs, IRs, GRs, SNMPs, OBPs and CSPs), the available sequences of ORs, IRs, GRs, SNMPs, OBPs and CSPs proteins from other insect species were used as queries. Related sequences were obtained by searching NCBI databases with keywords “odorant receptor AND insecta”, “ionotropic receptor OR ionotropic glutamate receptor AND insecta”, “gustatory receptor AND insecta”, “sensory neuron membrane protein AND insecta”, “odorant-binding protein AND insecta” and “chemosensory proteins AND insecta”). The retrieved queries were used to blast against our transcriptomes using tBLASTn with e-value cut-off <1e− 5. Subsequently, all identified candidate unigenes were manually checked by BLASTx searches against NCBI Nr database (e-value <1e-5). The ORFs (Open reading frames) of candidate chemosensory genes were predicted in the ORF finder tool at the NCBI (https://www.ncbi.nlm.nih.gov/orffinder/). Protein domains (e.g. transmembrane domains, signal peptides, secondary structures, etc.) were predicted by queries against InterPro using the InterProScan Geneious software plugin by running batches of analyses (e.g. HMMPanther, SignalPHMM, HMMPfam, TMHMM, HMMSmart, Superfamily, etc.). *B. minax* transcripts deemed orthologous (based on sequence similarity) to *D. melanogaster* sequences were given the same name (e.g. DmelIR15a, BminIR15a, DmelORco, BminORco). Multiple copies of a putative *D. melanogaster* orthologue were given the same name followed by a point and number (e.g. BminOR43a.1, BminOR43a.2, BminOR43a.3, and BminOR43a.4).

### Differential gene expression

The expression levels of these unigenes were calculated using FPKM method [71], which calculated by RSEM (Version: v1.3.0) with default parameters [72]. Differential gene expression in samples was measured using the DEGseq R package (Version: 1.12.0). DEGseq provides statistical routines for determining differential digital gene expression. *P*-value was adjusted using q-value with q < 0.05 & |log2(foldchange)| > 1 as the threshold for significantly differentially expressed genes. Heatmaps of gene expression for different chemosensory genes among female antennae, male antennae, female rectal glands and male rectal glands were generated by R version 3.4.1.

### Phylogenetic analysis

The amino acid sequences of predicted ORs, IRs, GRs, SNMPs, OBPs and CSPs from *B. minax* were aligned together with proteins from Dipterans with ClustalW method [73], and Maximum-likelihood trees were constructed in IQ-TREE (version 2.1.7) using best-fitting substitution-model [74]. Branch support was assessed with 1000 bootstrap replicates. Phylogenetic trees were visualized with FigTree (http://tree.bio.ed.ac.uk/software/figtree). OR sequences were obtained from *D. melanogaster*, *B. dorsalis*, *Calliphora stygia* and *M. domestica*. The IR data set contained IR sequences from *D. melanogaster*, *C. stygia*, and *C. capitate*. The GR data set contained GR sequences from *D. melanogaster*, *B. dorsalis*, and *C. stygia*. The OBP data set contained OBP sequences from *D. melanogaster*, *B. dorsalis*, *Anastrepha fraterculus*, *A. obliqua* and *C. capitate*. The SNMP data set contained SNMP sequences from *D. melanogaster*, *B. dorsalis*, *M. domestica* and *Anopheles gambiae*. For the CSP data set, we selected CSP sequences from *D. melanogaster*, *B. dorsalis*, *M. domestica* and *Glossina morsitans morsitans*. These sequences used for constructing phylogenetic trees are listed in Additional file 8: Table S5.

### Expression analysis by real-time quantitative PCR

The expression profiles of all 40 ORs and 14 antennal IRs were analyzed using RT-qPCR. Total RNA isolated from antennae (300 pairs of each sex), rectal glands (50 of each sex), foreleg tarsi (300 pairs of each sex), proboscises (50 of each sex) and wings (50 of each sex), and cDNA was synthesized using PrimeScrip RT Master Mix kit (Takara, China). RT-qPCR experiments including negative controls (without cDNA template) were performed on a Light Cycler 480 System (Roche Applied Science) using a mixture of 10 μl 2× SYBR Green PCR Master Mix, with the reaction programs: 95 °C for 15 min, followed by 40 cycles of 95 °C for 10 s and 60 °C for 32 s. Then, the PCR products were heated to 95 °C for 15 s, cooled to 60 °C for 1 min, heated to 95 °C for 30 s and cooled to 60 °C for 15 s to measure the dissociation curves. Each sample had three biological replicates and each replicate had three technical duplicates. Relative transcript abundance was determined using the 2^-ΔΔCT^ method [75]. To normalize the gene expression studies, two reference genes, α-tubulin (Bminα-TUB) and glyceraldehyde-3-phosphate dehydrogenase 2 (BminGAPDH2) were selected in our transcriptomes [76, 77]. Gene-specific primers were designed using Primer3 (http://primer3.ut.ee/) and are listed in Additional file 9: Table S6. Comparative analyses for each gene among various samples were analyzed with a one-way nested analysis of variance (ANOVA), followed by Tukey’s honestly significance difference (HSD) tests implemented in Prism 7.0 (GraphPad Software, CA). All values are presented as the mean ± SE.

## Additional files

Additional file 1: **Table S1.** Overview of the sequencing and assembly process. (XLSX 9 kb)

Additional file 2: **Table S2.** Functional annotation of the unigenes in different databases. (XLSX 10 kb)

Additional file 3: **Figure S1.** Results of BLASTx matches of *Bactrocera minax* transcriptome unigenes, Gene ontology classification and KEGG pathway annotation. A: insect species in which homologous genes were matched. B: Gene ontology classifications of *B. minax* unigenes. C: KEGG pathway annotation of *B. minax* unigenes. (TIF 2126 kb)

Additional file 4: **Table S3.** Candidate chemosensory genes in *Bactrocera minax*. Candidate ORs (sheet 1), IRs (sheet 2), GRs (sheet 3), SNMPs (sheet 4), OBPs (sheet 5), CSPs (sheet 6) and with gene name, predicted protein sequences, and the annotation in NCBI-Nr database, predicted protein domains and expression abundance. (XLSX 68 kb)

Additional file 5: **Figure S2.** Amino acid alignments of *Bactrocera minax* OBPs. Cysteines are indicated by red frames. The cysteines position are marked at the base. (TIF 5058 kb)

Additional file 6: **Figure S3.** Amino acid alignments of *Bactrocera minax* CSPs. Cysteines are indicated by red frames. The cysteines position are marked at the base. (TIF 395 kb)

Additional file 7: **Table S4.** FPKM value of candidate chemosensory genes in *Bactrocera minax*. (XLSX 22 kb)

Additional file 8: **Table S5.** GenBank accession numbers of chemosensory genes used in phylogenetic analyses. (XLSX 231 kb)

Additional file 9: **Table S6.** Primers of candidate ORs and antennal IRs in *Bactrocera minax* used for RT-qPCR. (XLSX 13 kb)

## Data Availability

The raw reads of the four transcriptomes in this study have been stored in the NCBI SRA database, under the accession number of SAMN10678451 (female antennae), SAMN10678452 (male antennae), SAMN10678453 (female rectal glands), and SAMN10678454 (male rectal glands).

## Abbreviations

ANOVA: Analysis of variance
CL: Cue lure
COG: Clusters of Orthologous Groups
CSPs: Chemosensory proteins
FPKM: Fragments per kilobase per million reads
GO: Gene Ontology
GRs: Gustatory receptors
HSD: Honestly significance difference
IRs: Ionotropic receptors
KEGG: Kyoto Encyclopedia of Genes and Genomes
ME: Methyl eugenol
OBPs: Odorant-binding proteins
ORco: OR co-receptor
ORFs: Open reading frames
ORNs: Olfactory receptor neurons
ORs: Odorant receptors
RK: Raspberry ketone
RT-qPCR: Real-time quantitative PCR
SE: Standard error
SNMPs: Sensory neuron membrane proteins
TMDs: Transmembrane domains
