# Supplementary figures and images for: Candidates for chemosensory genes identified in the Chinese citrus fly, Bactrocera minax, through a transcriptomic analysis
Source: BMC Genomics. 2019 Aug 14;20:646. doi: 10.1186/s12864-019-6022-5 (PMC6693287; doi:10.1186/s12864-019-6022-5)

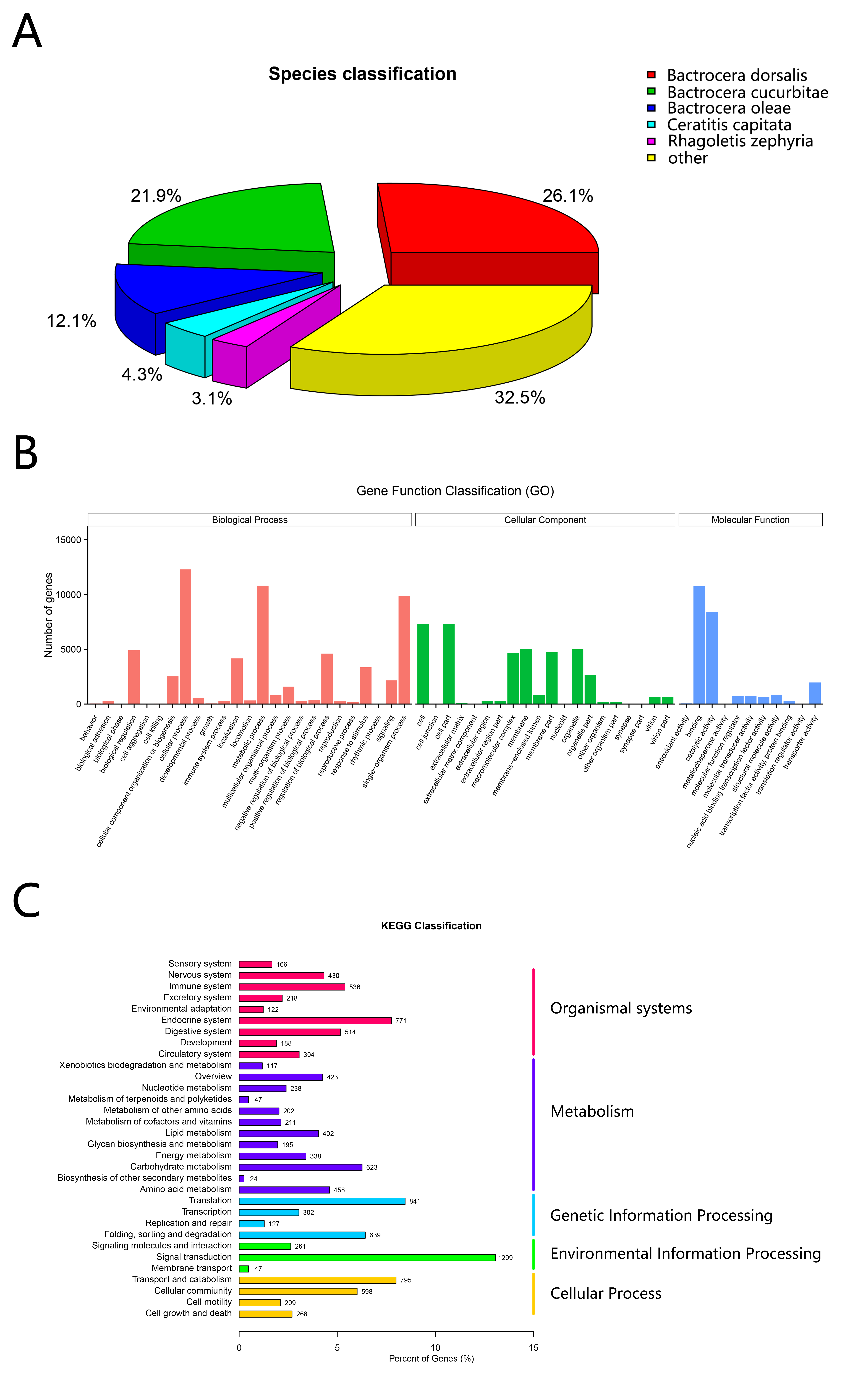

Supplement: Supplementary file 3 — Figure S1. Results of BLASTx matches of Bactrocera minax transcriptome unigenes, Gene ontology classification and KEGG pathway annotation. A: insect species in which homologous genes were matched. B: Gene ontology classifications of B. minax unigenes. C: KEGG pathway annotation of B. minax unigenes. (TIF 2126 kb) [file 12864_2019_6022_MOESM3_ESM.tif]

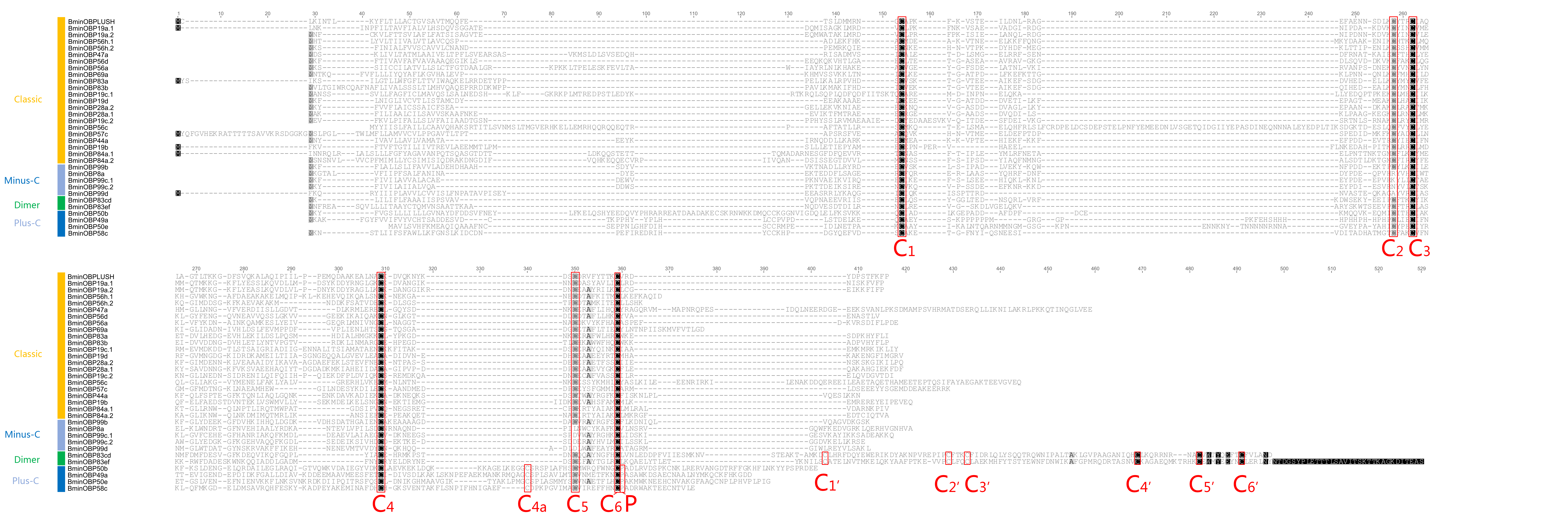

Supplement: Supplementary file 5 — Figure S2. Amino acid alignments of Bactrocera minax OBPs. Cysteines are indicated by red frames. The cysteines position are marked at the base. (TIF 5058 kb) [file 12864_2019_6022_MOESM5_ESM.tif]

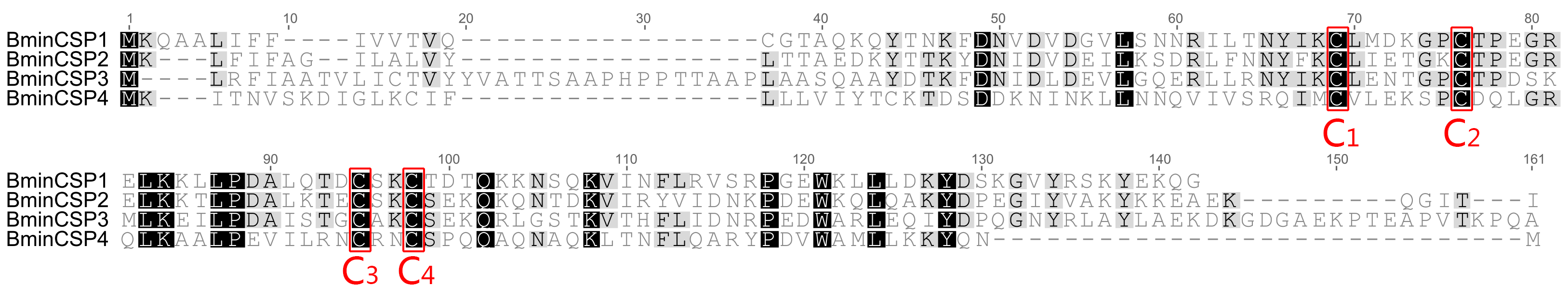

Supplement: Supplementary file 6 — Figure S3. Amino acid alignments of Bactrocera minax CSPs. Cysteines are indicated by red frames. The cysteines position are marked at the base. (TIF 395 kb) [file 12864_2019_6022_MOESM6_ESM.tif]
